# Survey of bovine fasciolosis burdens in trade cattle slaughtered at abattoirs in North-central Nigeria: The associated predisposing factors and economic implication

**DOI:** 10.1016/j.parepi.2017.02.001

**Published:** 2017-02-22

**Authors:** Suleiman Yatswako, Nma Bida Alhaji

**Affiliations:** a Epidemiology Division, Niger State Ministry of Livestock and Fisheries Development, Minna, Nigeria; b Department of Veterinary Public Health and Preventive Medicine, University of Ibadan, Ibadan, Nigeria

## 1 Introduction

Fasciolosis, transmitted by fresh water snails, *Lymnae*, is an important parasitic zoonotic disease caused by liver flukes of the genus *Fasciola*, with two important species, *Fasciola hepatica* and *F. gigantic* (Andrews, 1999, Mas-Coma et al., 2005). Fasciolosis has the largest geographical widespread of any emerging vector-borne zoonosis, occurring in > 51 countries worldwide. The complex nature of the epidemiology of this snail-borne disease presents challenges for disease management and animal husbandry (Mas-Coma et al., 2009). Gretera et al. (2016) reported a baseline bovine fascioliasis prevalence of 41.9% and higher prevalence of 46.0% at 6-month post-treatment follow-up at the Lake Chad, with increment associated with re-infection due to mobile animal husbandry system. *Fasciola hepatica* is mainly distributed in temperate regions such as Europe, the Americas, and Australia, with limited distribution of its intermediate host *Lymnaea* (*Galba*) *truncatula*, while *F. gigantic* is more prevalent in tropical countries being adapted to warmer conditions likely due to the wide distribution of its intermediate host *Lymnaea* (*Radix*) *natalensis*, and both species have been found in sub-Saharan Africa and Asia (Walker et al., 2008, Mas-Coma et al., 2009, Kanyari et al., 2010).

*Fasciola* has a two-host life cycle. Its asexual stage develops in the intermediate hosts, which in nature are mostly freshwater snails, *Lymnaea* spp. and the sexual stage is in cattle and other ruminants, which are the definitive hosts. Animals got infected by eating forage contaminated with the metacercariae of the flukes. They can also be infected with ingestion of cysts suspended in soil and detritus while drinking water. Ingested parasite finds its way into intra hepatic biliary duct or hepatic parenchyma and later to the bile duct where it resides (Andrews, 1999, Bargues and Mas-Coma, 2005, Mas-Coma et al., 2009).

The major source of loss to domestic animal production in Africa, Asia, Tropical and Sub-tropical areas has been traced to fasciolosis (Hammond, 1965). Fasciolosis causes significant economic losses to global agriculture, estimated at > 3 billion USD annually, through liver condemnation and reduction of milk and meat yields (Khaitsa et al., 1994, Mas-Coma, 1997, Biu et al., 2006, Abunna et al., 2010). Furthermore, the disease is of great public health concern because of an increasing number of reported human cases from accidental ingestion of *Fasciola* eggs/larvae (WHO, 1995, Mas-Coma et al., 2009).

Abattoir surveillance has been used in many countries as an important strategy for detection of disease cases and provides essential information that can be utilized for research and disease control purposes (Phiri, 2006). The use of meat inspection to detect disease cases in slaughter facilities is particularly useful in Africa where laboratory capacity for routine disease diagnosis is limited (Phiri, 2006, Cadmus and Adesokan, 2009). With paucity of empirical information on the burdens and associated predisposing internal (biological) and external (climatic and environmental) factors for fasciolosis, such data are needed to serve as convenient and inexpensive source of information for the development of fasciolosis control programs in Africa. This study was, therefore, aimed to investigate burdens, associated risks and economic impact of bovine fasciolosis in trade cattle slaughtered in municipal abattoirs of North-central Nigeria. Our Null hypothesis was that biological characteristics of animals (intrinsic factors), seasons and geographical locations (extrinsic factors) cannot influence occurrence of bovine fasciolosis in Nigeria.

## 2 Materials and methods

### 2.1 Study area

**Fig. 1:**
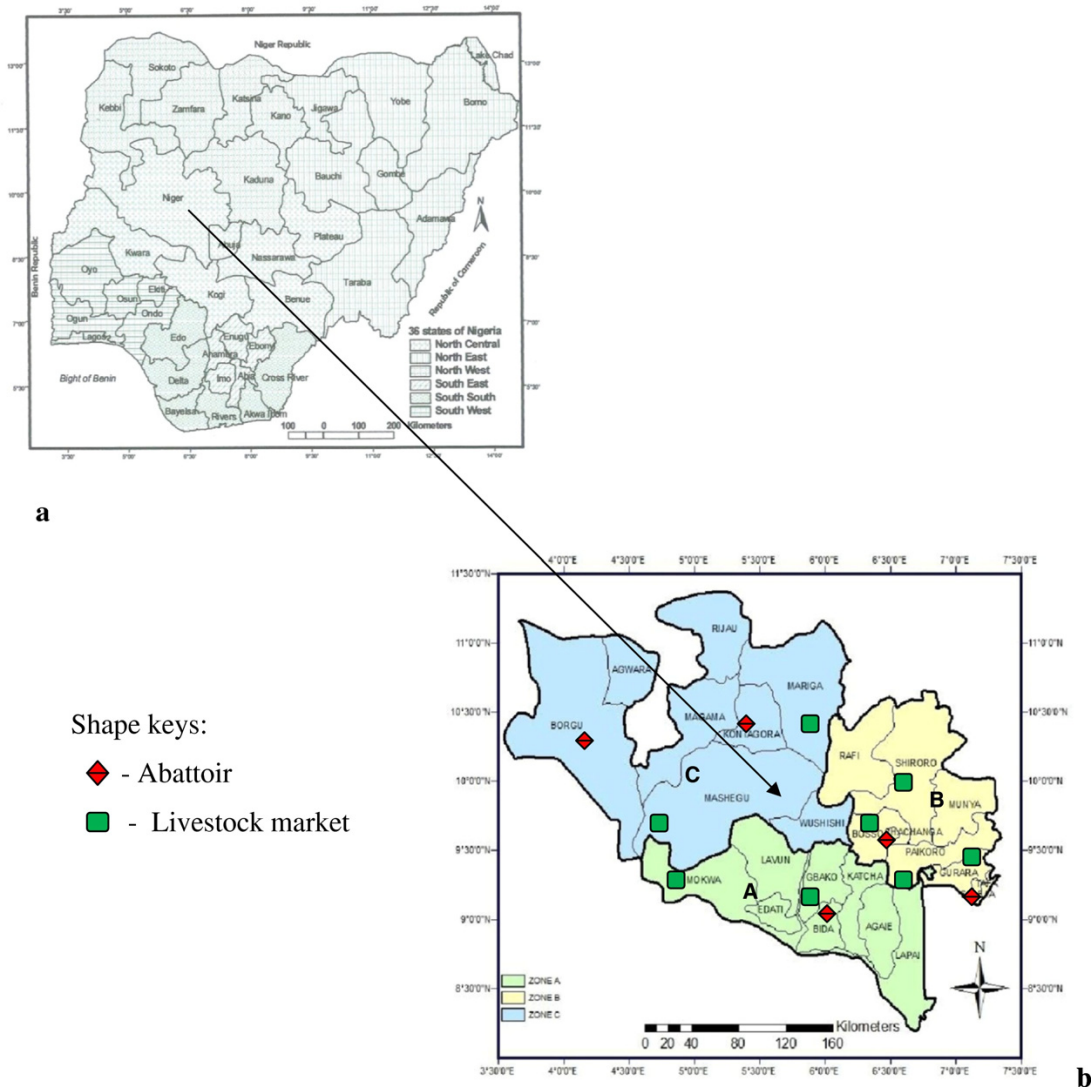
1a: Map of Nigeria showing the location of Niger State. 1b: Map of Niger State showing the three Agro-geographical zones A, B and C in the state with their LGAs.

The study was conducted in Niger State, located at the North-central geopolitical zone of Nigeria, with geographical coordinates of latitude 8° 20′ N and 11° 30′ N, and longitude 3° 30′ E and 7° 20′ E. It is one of the 36 states and the largest in terms of land mass, covering an area of 86,000 km^2^, representing about 9.3% of the total land area of the country. Niger State has three Agro-geographical zones, with variable climatic conditions. These are: Agro-geographical zone A (Southern zone), with eight local governments areas (LGAs), many rivers, streams and ponds, fadamas for rice farming and large grazing lands; Agro-geographical zone B (Eastern zone), with nine LGAs, many mountains, trees, and few rivers and streams, arable and grazing lands; and Agro-geographical zone C (Northern zone), with eight LGAs, few rivers and streams, arable and large grazing areas, and many stock routes (Fig. 1).

The State experiences four distinct seasons: early dry season (October–December), late dry season (January–March), early rainy season (April–June), and late dry season (July–September). The mean annual rainfall is 1600 mm with duration of about 180 days. It has humidity of 104% and average lowest and highest temperatures of about 27 °C and 39 °C, respectfully. It provides transit routes for pastoral nomads on seasonal migrations from the northern parts to the southern parts of Nigeria. In 2006, the human population in the state was estimated at 3.94 million people (NPC, 2006). According to the Nigerian Livestock Resources Survey, the state has an estimated cattle population of 2.4 million cattle in 2012, which are mostly infected by *Fasciola* spp. and are in the custodies of nomadic and sedentary pastoralists (MLFD, 2015).

### 2.2 Study design and target populations

Retrospective and prospective abattoir surveys were conducted at five municipal abattoirs, located at Minna, Suleja, Bida, Kontagora and New-Bussa cities. The retrospective survey was carried out between January 2004 and December 2014, and secondary data were retrieved from the meat inspections records of slaughtered trade cattle at the abattoirs. The prospective study was conducted at the five abattoirs between January 2015 and December 2015, and bile samples were collected at post-mortem inspections from gall bladders of slaughtered the animals.

Study populations were trade cattle that originated from nomadic and sedentary pastoral herds domiciled in the three Agro-geographical zones. They were aged one year and above, of both sexes (bulls and cows) and available breeds (Bunaji, Bokologi and Rahaji) brought from livestock markets to the abattoirs and slaughtered. Animals purchased records indicated that they were bought at Jebba, Wuya, Lambata, Tunga-Mallam, Kuta, Beji, Mariga and Zugurma livestock markets, which are located in the three agro-geographical zones of the state. However, animals were usually allowed to rest for at least 24 h before slaughtered.

### 2.3 Sample size determination and sampling method

Sample size was determined only for the prospective study. Because of the inconsistency in the number of daily slaughtered trade cattle, sample size was calculated for proportion of infinite population using the Open Source Epidemiologic Statistics for Public Health OpenEpi 2.3 software (Dean et al., 2009), with power set at 50% and 5% margin of error at 95% confidence level. A sample size of 384 animals was obtained for each abattoir, and a total of 1920 cattle were selected from the five abattoirs during the one year survey. The abattoirs, being major slaughterhouses, were purposively selected across the study area. Systematic random sampling method was used to select the slaughtered animals at sampling interval of two.

The study protocol was approved by the Niger State Ministry of Livestock and Fisheries Development Research Ethics Committee. Advocacy visits were made to the management of each abattoir prior to the time of samples collection.

### 2.4 Sample collection and laboratory analysis

Liver inspection was carried out by visual examination, palpation and incision of the organ. *Fasciola* infection was judged based on liver enlargement with bumpy, raised and/or depressed areas, dark blue to black discolorations, hardness in consistence and during incision when liver flukes were seen with morphological structures of flat bodies, oval shapes and suckers on the ventral sides.

During the prospective survey period, 2 ml of bile sample was collected from gall bladder of each of the 1920 sampled slaughtered cattle, and 2 ml sterile syringe was used for each animal. Each bile sample was poured into a labeled test tube in a test tube rack, and 1 ml of 10% formalin was added into the bile sample and allowed to stand for 5 min. Also, 1 ml diethyl-ether was added into the test tube after 5 min. The content in the test tube was then corked, shaken and the solution mixed. The solution was then centrifuged at 2000 rpm for 10 min and the eggs of *Fasciola* settled at bottom of the tube, while diethyl-ether with some fats suspended as supernatant. The supernatant was decanted and the sediment left in the test tube. One to two drops of the sediment were put on a glass slide, covered with a slip and viewed under microscope using 100 × magnifications (Cheesbrough, 1999). A sample was considered positive if a *Fasciola e*gg with the correct morphology of ellipsoidal and operculated structure was observed (Valero et al., 2009).

### 2.5 Data management and statistical analyses

Collected data were summarized into Microsoft Excel 7 spreadsheet (Microsoft Corporation, Redmond, WA, USA) and the OpenEpi version 2.3.1 software (Dean et al., 2009) was used for analysis. Descriptive statistics of proportions were used to describe some of the obtained data.

Associations between the predisposing factors and occurrence of bovine fasciolosis were assessed. Intrinsic cattle characteristics of age, sex, and breed as well as extrinsic seasonal and geographical location factors constituted the covariates (hypothesized independent or explanatory) variables, while those cattle with and without fasciolosis cases constituted the dependent (outcome) variables. The associations between explanatory factors and outcome variables were first subjected to univariate analysis using Chi-square tests (Dohoo et al., 2009). All factors found to be biologically plausible and significant were finally subjected to multivariate analyses using Likelihood stepwise backward logistic regression models to control for confounding and test for effect modification. The Hosmer and Lemeshow test was used to assess for goodness of fit of the final model and was found to be good. P < 0.05 was considered statistically significant at all analyses.

### 2.6 Economic loss assessment due to liver condemnation

The total economic loss was calculated from the summation of livers condemned from 2005 to 2014 with available complete data as well as those condemned in 2015. The estimated costs were considered according to Nigerian naira (NGN) exchange rate to the US dollar (USD) in 2015. The total economic loss due to *Fasciola* infected livers condemned was calculated using the formula:

TEL=N∗P∗W

where: *TEL*, is total economic loss; *N*, is total number of condemned livers; *P*, is average liver price (dollar/kg); and *W*, is average liver weight (kg). The average weight of cattle livers was 3.2 kg, obtained from a pilot study in abattoirs by weighting 1317 healthy livers from slaughtered cattle. The average sell price for each kilogram of liver was 5.0 USD (980 NGN), acquired by interviewing local butchers and meat sellers in the study area. The exchange rate of 196.99 NGN to 1 USD, available at the time of survey was used (CBN, 2015).

## 3 Results

### 3.1 Burden of bovine fasciolosis from retrospective records of condemned livers

**Fig. 2:**
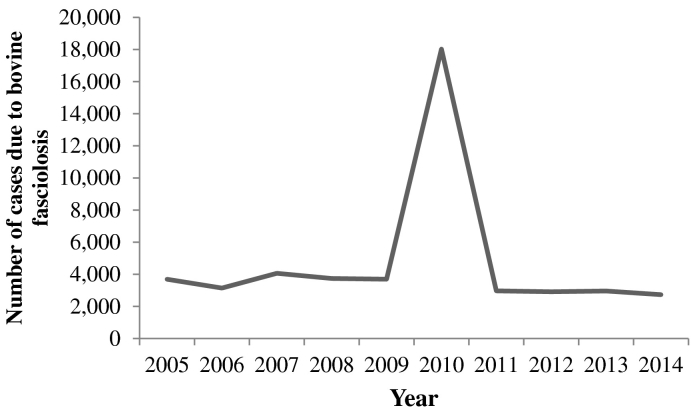
Annual trend of bovine fasciolosis cases in slaughtered trade cattle at five municipal abattoirs in North-central Nigeria: 2005–2014.

**Fig. 3:**
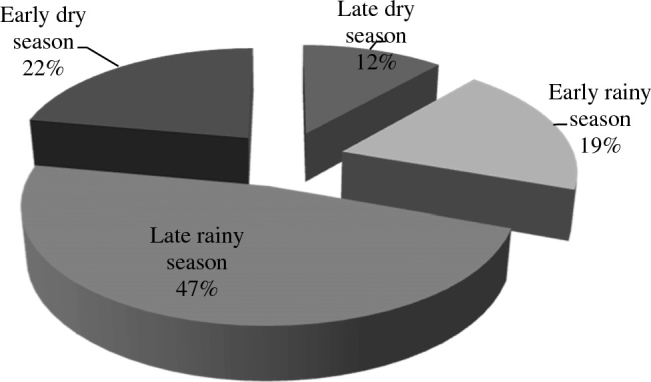
Proportion of seasonal condemnation of livers at post-mortem inspections due to bovine fasciolosis at five municipal abattoirs in North-central Nigeria: 2005–2014.

From the retrospective survey, a total of 3,292,634 trade cattle were slaughtered and inspected at Minna, Suleja, Bida, Kontagora and New-Bussa municipal abattoirs during the period 2005 to 2014 (Table 1). Of the slaughtered cattle, 47,931 had their livers condemned due to pathological conditions indicative of fasciolosis (Table 2). The observed prevalence was 1.31% (95% CI: 1.28–1.34), 1.70% (95% CI: 1.68–1.72), 1.11% (95% CI: 1.09–1.15), 1.05% (95% CI: 1.02–1.08), and 1.24% (95% CI: 1.20–1.29) at Minna, Suleja, Bida, Kontagora and New-Bussa abattoirs, respectively. The overall ten-year prevalence of bovine fasciolosis was 1.46% (95% CI: 1.44–1.47) (Table 3). The annual trend of bovine fasciolosis cases in slaughtered trade cattle and proportions of seasonal condemnations of livers at post-mortem due to the disease at the municipal abattoirs is presented in Fig. 2, Fig. 3, respectively.

**Table 1:** Annual slaughtered trade cattle at five municipal abattoirs in North-central Nigeria: 2005–2014.

Abattoir	2005	2006	2007	2008	2009	2010	2011	2012	2013	2014	Total
Minna	34,658	45,940	41,852	42,921	34,184	37,208	38,228	89,782	129,999	44,174	538,946
Suleja	103,704	137,977	127,596	131,671	119,552	121,278	133,846	301,418	389,979	148,642	1,715,663
Bida	30,292	42,437	35,763	33,286	29,067	31,334	31,052	49,609	94,967	56,793	434,600
Kontagora	26,268	38,139	32,168	31,994	30,621	31,169	23,789	35,798	49,971	45,107	345,024
New Bussa	21,416	19,845	19,947	23,056	23,965	22,789	23,465	29,100	35,763	39,055	258,401
Total	216,338	284,338	257,326	262,928	237,389	243,778	250,380	505,707	700,679	333,771	3,292,634

**Table 2:** Annual cases of bovine fasciolosis from condemned livers of slaughtered trade cattle at five municipal abattoirs in North-central Nigeria: 2005–2014.

Abattoir	2005	2006	2007	2008	2009	2010	2011	2012	2013	2014	Total
Minna	785	701	819	836	779	597	652	601	670	640	7080
Suleja	1538	1188	1751	1647	1731	16,671	1165	1109	1265	1107	29,172
Bida	578	533	673	584	499	379	471	423	312	402	4854
Kontagora	411	432	498	297	369	158	346	418	381	307	3617
New-Bussa	378	290	321	377	316	223	335	362	331	275	3208
Total	3690	3144	4062	3741	3694	18,028	2969	2913	2959	2731	47,931

**Table 3:** Burdens of bovine fasciolosis from condemned livers of slaughtered trade cattle at five municipal abattoirs in North-central Nigeria: 2005–2014.

Abattoir	Total slaughtered cattle	Total cases	Burden (%)	95% CI
Minna	538,946	7080	1.31	1.28–1.34
Suleja	1,715,663	29,172	1.70	1.68–1.72
Bida	434,600	4854	1.11	1.09–1.15
Kontagora	345,024	3617	1.05	1.02–1.08
New-Bussa	258,401	3208	1.24	1.20–1.29
Total	3,292,634	47,931	1.46	1.44–1.47

### 3.2 Prevalence of bovine fasciolosis during prospective survey of bile

**Fig. 4:**
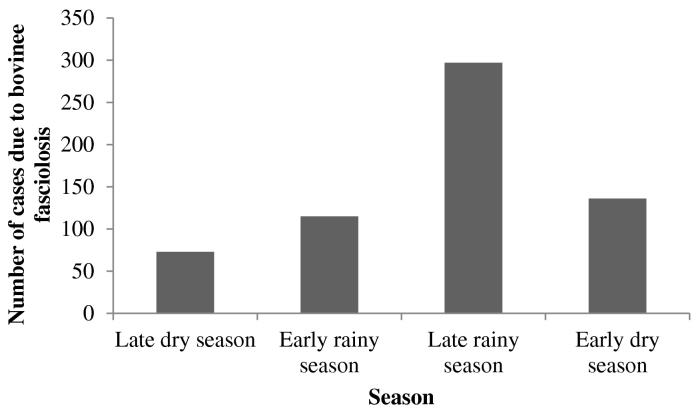
Seasonal cases of bovine fasciolosis from bile of infected slaughtered trade cattle at five municipal abattoirs in North-central Nigeria: January to December 2015.

Using bile samples from slaughtered cattle, 621 out of 1920 slaughtered cattle bile examined had *Fasciola* eggs and affected livers were condemned. The prevalence in Minna abattoir was 32.29% (95% CI: 27.75, 37.1), Suleja was 26.82% (95% CI: 22.46, 31.42), Bida was 30.47% (95% CI: 26.02, 35.21), Kontagora was 35.42% (95% CI: 30.75, 40.3), and New-Bussa had 36.72% (95% CI: 32.0, 41.63). However, the overall prevalence was 32.34% (95% CI: 30.28, 34.46) (Table 4). Seasonal cases of bovine fasciolosis from bile of infected slaughtered trade cattle at the municipal abattoirs are presented in Fig. 4.

**Table 4:** Burdens of bovine fasciolosis from bile samples of slaughtered trade cattle at five municipal abattoirs in North-central Nigeria: January to December 2015.

Abattoir	Number of cattle sampled	Number positive	Number negative	Burden (%)	95% CI
Minna	384	124	260	32.29	27.75, 37.1
Suleja	384	103	281	26.82	22.46, 31.42
Bida	384	117	267	30.47	26.02, 35.21
Kontagora	384	136	248	35.42	30.75, 40.3
New-Bussa	384	141	243	36.72	32.0, 41.63
Total	1920	621	1299	32.34	30.28, 34.46

### 3.3 Intrinsic and extrinsic factors associated with occurrence of bovine fasciolosis during retrospective survey

During retrospective study, breed and age were found to significantly influenced occurrence of bovine fasciolosis at the univariable analysis. In the final multivariable logistic regressions, Bokoloji (Sokoto gudali) breed was less likely (OR 0.79; 95% CI: 0.78, 0.82) to be significantly predisposed to *Fasciola* infection as Rahaji (Red bororo) breed. However, Bunaji (White bororo) breed was more likely (OR 1.04; 95% CI: 1.01, 1.06) to be significantly predisposed to the infection than Rahaji breed. Also, cattle aged ≥ 3 were less likely (OR 0.26; 95% CI: 0.25, 0.26) to be significantly predisposed to *Fasciola* infection as those in age group 1–3 years (Table 5). As for the extrinsic factors, early rainy and late rainy seasons were more likely [(OR 1.45; 95% CI: 1.40, 1.50) and (OR 2.32; 95% CI: 2.25, 2.39), respectively] to significantly influenced occurrence of bovine fasciolosis than late dry season. Also, early late season was two times more likely (OR 1.69; 95% CI: 1.64, 1.76) to significantly influenced occurrence of bovine fasciolosis. Furthermore, Agro-geographical zone B was more likely (OR 1.20; 95% CI: 1.17, 1.23) to possessed geographical features that significantly predisposed cattle to *Fasciola* infections as Agro-zone A; while Agro-zone C was less likely (OR 0.84; 95% CI: 0.81, 0.87) to have geographical features that significantly predisposed to cattle to the infections as Agro-zone A (Table 5).

**Table 5:** Multivariate logistic regressions for intrinsic and extrinsic factors associated with livers condemnation due to bovine fasciolosis in slaughtered trade cattle at five municipal abattoirs in North-central Nigeria: 2005–2014.

Factor	Cattle without fasciolosis (Row %)	Cattle with fasciolosis (Row %)	Odds ratio (95% CI)	P-value
Breed				
Rahaji	533,826 (98.5)	8099 (1.5)	1.00	
Bokoloji	1,066,554 (98.8)	12,920 (1.2)	0.79 (0.78, 0.82)	< 0.001⁎
Bunaji	1,645,323 (98.4)	25,912 (1.6)	1.04 (1.01, 1.06)	0.003⁎

Age				
1–3	1,178,735 (97.3)	32,954 (2.7)	1.00	< 0.001⁎
≥ 3	2,065,969 (98.3)	14,977 (0.7)	0.26 (0.25, 0.26)	

Season				
Late dry season	659,456 (99.1)	5656 (0.8)	1.00	
Early rainy season	712,325 (98.8)	8862 (1.2)	1.45 (1.40, 1.50)	< 0.001⁎
Late rainy season	1,152,554 (98.1)	22,916 (1.9)	2.32 (2.25, 2.39)	< 0.001⁎
Early dry season	720,368 (98.6)	10,497 (1.4)	1.69 (1.64, 1.76)	< 0.001⁎

Agro-geographical zone				
Zone A	429,746 (98.9)	4854 (1.1)	1.00	
Zone B	2,218,357 (98.4)	36,252 (1.6)5	1.20 (1.17, 1.23)	< 0.001⁎
Zone C	596,600 (98.9)	6825 (1.1)	0.84 (0.81, 0.87)	< 0.001⁎

⁎ Significant at P < 0.05.

### 3.4 Intrinsic and extrinsic factors associated with occurrence of bovine fasciolosis at prospective study

At the prospective study, all intrinsic factors of breed, sex and age significantly influenced occurrence of the disease at univariable analysis. However, during multivariable logistic regressions, only Bunaji breed was more likely (OR 1.32; 95% CI: 1.03, 1.79) to be significantly predisposed to *Fasciola* infection than Rahaji breed. Also, cows were more likely (OR 1.86; 95% CI: 1.49, 2.19) to be significantly predisposed to *Fasciola* infection than the bulls. Further, cattle aged ≥ 3 were less likely (OR 0.28; 95% CI: 0.23, 0.35) to be significantly predisposed to *Fasciola* infection as those in age group 1–3 years. As for the extrinsic factors, early rainy and early dry seasons were more likely [(OR 1.76; 95% CI: 1.27, 2.43) and (OR 2.20; 95% CI: 1.60, 3.03), respectively] to significantly influenced occurrence of bovine fasciolosis than late dry season. And late rainy season was nine times more likely (OR 9.05; 95% CI: 6.64, 12.33) to significantly influenced occurrence of the disease than late dry season. Also, only Agro-geographical zone C was more likely (OR 1.29; 95% CI: 0.99, 1.67) to possessed geographical features that significantly predisposed cattle to *Fasciola* infections than Agro-zone A (Table 6).

**Table 6:** Multivariate logistic regressions for intrinsic and extrinsic factors associated with occurrence of bovine fasciolosis in bile of slaughtered trade cattle at five municipal abattoirs in North-central Nigeria: January to December 2015.

Factor	Cattle without fasciolosis (Row %)	Cattle with fasciolosis (Row %)	Odds ratio (95% CI)	P-value
Breed				
Rahaji	261 (70.2)	111 (29.8)	1.00	
Bokoloji	377 (73.2)	138 (26.8)	0.86 (0.64, 1.57)	0.320
Bunaji	661 (64.0)	372 (36.0)	1.32 (1.03, 1.79)	0.030⁎

Sex				
Bulls	711 (74.1)	249 (25.9)	1.00	
Cows	588 (61.2)	372 (38.8)	1.86 (1.49, 2.19)	< 0.001⁎

Age				
1–3	241 (46.6)	276 (53.4)	1.00	
≥ 3	1058 (75.4)	345 (24.6)	0.28 (0.23, 0.35)	< 0.001⁎

Season				
Late dry season	407 (84.8)	73 (15.2)	1.00	
Early rainy season	365 (76.0)	115 (24.0)	1.76 (1.27, 2.43)	0.001⁎
Late rainy season	183 (38.1)	297 (61.9)	9.05 (6.64, 12.33)	< 0.001⁎
Early dry season	344 (71.7)	136 (28.3)	2.20 (1.60, 3.03)	0.001⁎

Agro-geographical zone				
Zone A	267 (69.5)	117 (30.5)	1.00	
Zone B	541 (70.4)	227 (29.6)	0.96 (0.73, 1.25)	0.748
Zone C	491 (64.0)	277 (36.0)	1.29 (0.99, 1.67)	0.050⁎

⁎ Significant at P < 0.05.

### 3.5 Total economic loss

From the retrospective survey, the total economic loss was calculated by multiplying the total number of the condemned livers due to fasciolosis within the ten year period (2005 to 2014) by average price (5.0 USD) per kilogram weight of healthy fresh liver and value of average weight (3.2 kg) of liver. An estimated total economic loss of 766,896.0 USD was obtained from 47,931 livers condemned. As for the prospective study, 631 condemned livers in 2015 resulted in an estimated economic loss of 9936 USD. However, the overall total economic loss incurred between 2005 and 2015 from condemned 48,552 livers in the five abattoirs was estimated at 776,832 USD.

## 4 Discussion

Abattoir is important in the supply of safe meat and meat products for human consumption and also for surveillance of animal and zoonotic diseases. The present research provides information on the burdens of natural *Fasciola* infections in the definitive host (cattle) at five municipal abattoirs in North-central Nigeria. The overall retrospective prevalence of bovine fasciolosis during a ten-year period was 1.46%. Higher five-year bovine fasciolosis burden of 14.6% from condemned livers has been reported at Makurdi abattoirs in Nigeria (Ejeh et al., 2015). Also, a higher 28.0% level of fluke infection has been recorded at an abattoir in northern Portugal/Spain (Arias et al., 2011), and much higher prevalence of 68.0% from the disease has been reported in slaughtered cattle at the Lake Chad (Jean-Richard et al., 2014). The discrepancies in the disease proportions may be attributed to different intrinsic and extrinsic factors associated with the animals, period of time covered, volumes of throughputs and operating standard procedures in the abattoirs. There was peak of cases in 2010, which could be due to very high rainfalls recorded in that year, with resultant flooding and formation of more swampy areas across the grazing fields that favored snails multiplications.

A prospective prevalence of 32.34% was observed in this study. This indicates that bovine fasciolosis is endemic in North-central Nigeria. The obtained prevalence is higher than the 27.7% reported in bile of cattle slaughtered at Sokoto Metropolitan Abattoir in Nigeria (Magaji et al., 2014). The differences in burdens could be due to variable climatic and ecological conditions such as rainfall, seasons, temperature and grazing areas. Climatic condition in the present study area favors high rainfall (MLFD, 2015), which in turn favors survival of the intermediate hosts (snails). The snails prefer swampy areas with slowly moving water and small streams, which allow sufficient moisture for the survival of the infective metacercariae. Ibironke and Fasina (2010) have previously reported that cattle livers are condemned at slaughterhouses in Nigeria due to fasciolosis than other diseases.

The biological parameters of breed, sex and age have significantly influenced susceptibilities of cattle to *Fasciola* infections in this survey. Influence of breed could be due to the differences in genetics, physiological, and immunological constitutions of the species. In our prospective study, cows have more infection burden (38.8%) than the bulls (25.9%). This is consistent with the reports of Ulayi et al. (2007) in Nigeria and Fatima and Chislti (2008) in Egypt that observed higher liver condemnations due to fasciolosis in cows than in bulls. Soulsby (1982) and Schillhorn van Veen (1997) have reported their studies that hormone-controlled relaxation of immunity in female animals during pregnancy and lactation are responsible for increases in their susceptibility to *Fasciola* infections. However, our finding is contrary to the reports of Idris and Madara (2005) and Obadiah (2010) that observed higher infection rates among the bulls than the cows slaughtered at Gwagwalada and Jalingo abattoirs in Nigeria, respectively. This present research also found age to have significant influence on occurrence of fasciolosis in cattle, which is more in young cattle (1–3 years old) than adult ones (≥ 3 years old). The reason could be due to the development of acquired immunity in the older animals that results in resistance, as opined by earlier investigators (Phiri et al., 2005).

In the present studies, seasons had significant influence on bovine fasciolosis occurrence among trade cattle. The burdens were more during the early rainy season, followed by late rainy season and starts falling at early dry season. These corroborate reports of Njoku-Tony (2011) indicate high rate of bovine fasciolosis in cattle slaughtered during the rainy season in Imo state, Nigeria. The high burdens at the rainy seasons are likely due to high volume of snails' availability at the grazing pastures and the fact that cattle acquired more infective stage of *Fasciola* while grazing at grazing fields, along river banks and around water bodies during the early rainy season and reaching peak at late rainy season. Grazing animals at seasonal extensions of rivers and lakes have been reported to predispose them to high risk of *Fasciola* infections (Jean-Richard et al., 2014). Our findings are also consistent with the reports of Damwesh and Ardo (2012) that found high prevalence of bovine fasciolosis at rainy season in Adamawa state, North-east Nigeria. Cattle acquire *Fasciola* infections during grazing at the onset of rainy season through ingestion of metacercariae (infective stage), when snail (*Lymnea* sp.) come out of their hibernation and release large numbers of metacercariae (Njoku-Tony, 2011, Ardo et al., 2013, Shahzad et al., 2014). Furthermore, Qureshi et al. (2012) have reported that metacercariae can be found on vegetation in large number during rainy season and at early dry season along river banks, lakes, and streams.

However, our investigations found low burdens fascilosis during the dry seasons*.* This is likely due to restricted concentration of snails along water bodies during dry season, which are rarely found in the dry grazing fields. Kuchai et al. (2011) have previously reported similar finding of low prevalence of bovine fasciolosis during dry season among cattle of Ladakh in Egypt. In contrast, high liver condemnations due to fasciolosis have been reported at dry season in Ethiopia and Nigeria, and were attributed to long prepatent period that enhance additional infections with resultant elevated prevalence of patent fasciolosis in the late rainy season, which might occur up to the dry season (Abdulhakim and Addis, 2012, Ejeh et al., 2015). The seasonal pattern of *Fasciola* infections represents an increased infection rates in the rainy season than in the dry season in our surveys, as previously reported (Sissay et al., 2007). Similar findings on seasonal influence on fasciolosis burden in Southern Espírito Santo and Punjab in Pakistan have also been reported (Bernardo et al., 2011, Qureshi et al., 2012, Shahzad et al., 2014).

Significant influence of geographical locations on bovine fasciolosis occurrence was observed in this research. The observed burdens might be due to suitability of geographical features that favor multiplication and survival of the intermediate hosts. Heavily infected cattle with *Fasciola* spp. with prevalence of up to 3.8% have been reported along riversides of the Niger River in Niger Republic, due to favorable humidity and temperature (Ali et al., 2008). Further, dwindling grazing lands availability during rainy seasons due to increased food crops farming, compelled animals on extensive system of management to graze in areas, such as riversides, that are heavily infested with snails in the seasons.

The overall total economic loss incurred between 2005 and 2015 from condemned 48,552 livers in this research was estimated at 776,832 USD. Similar economic losses have been reported from other parts of the globe. Ardo et al. (2013) have reported total economic loss of 9121.0 USD due to fasciolosis at major abattoirs in Adamawa State, North-east Nigeria. In Switzerland, Schweitzer et al. (2005) reported a loss of 42.8 million USD, while Regassa et al. (2012) reported economic loss of 13,364.72 USD in Central Ethiopia, all due to fasciolosis. These figures indicate that the disease is of high economic importance to the livestock industry.

From the available evidence, no design herd-based intervention is currently available for management of ruminant fasciolosis in Nigeria. However, future interventions based upon de-worming are clearly worthwhile in addition to the collection of local information on animal husbandry practices, economic impact and animal trafficking. The latter is especially important with future cattle re-stocking following the civil insecurities associated with cattle rustling in Nigeria. In terms of future disease surveillance on the detection of populations of *Lymnae* snails, it would be advisable to raise awareness of fluke-borne diseases in among pastoralists. The observed high burden of *Fasciola* eggs in bile shows that a single pre-rainy season treatment cannot prevent re-infection of herds at rainy season. An acceptable adapted strategic control package for fascioliasis in extensively managed cattle ought to integrate targeted anthelmintic treatment on seasonal movement practices.

The sensitivity of liver inspection at post-mortem has been reported to be 63–71% (Khaitsa et al., 1994). Also, a study by Rapsch et al. (2006) has indicated that meat inspection for liver fluke may exhibits a sensitivity of 63.2% (55.6–70.6%), meaning that the true levels of infection may be between 1.5 and 2 times the apparent prevalence. Although useful for the confirmation of patently infected animals, sensitivity of data from abattoir records in this research may not be optimal, which is a major limitation. However, the available evidences were substantiated in the light of our conjoined empirical coprological findings from the bile samples. We were also not being able to assign period of exposure to positive animals because of difficulty in trace back due to limited available technology and resources.

## 5 Conclusion

We have demonstrated that despite the limitations of using data from meat inspection records, they provide a significant cost-effective epidemiological resource that may have been underutilized. Both studies have established epidemiological understanding of biological, seasonal and environmental influences on the occurrence of bovine fasciolosis. The predictive values of *Fasciola* infections in slaughtered cattle were intrinsically and extrinsically defined and suggest levels of fasciolosis burdens in the herds. With the observed burdens of bovine fasciolosis and associated high economic implications, we recommend active herd-level surveillance and institution of effective control measures against the disease through application of molluscide drugs on grazing fields against the intermediate hosts, and well-defined seasonal interval for deworming of cattle at herd-levels before, during and immediately after the rainy season. Also, trade cattle should be dewormed at livestock markets with effective anthelmintics such as albendazole and praziquantel at least two weeks before slaughter; strict enforcement of meat inspection with good standard operating procedure (SOP); and good abattoir record keeping that provides information on livestock diseases are recommended.

## References

[bb0005] Abdulhakim Y., Addis M. (2012). An abattoir study on the prevalence of fasciolosis in cattle, sheep and goats in Debre Zeit town, Ethiopia. Global Vet..

[bb0010] Abunna F., Asfaw L., Megersa B., Regassa A. (2010). Bovine fasciolosis: coprological, abattoir survey and its economic impact due to liver condemnation at Soddo municipal abattoir, Southern Ethiopia. Trop. Anim. Health Prod..

[bb0015] Ali H., Ai L., Song H.Q., Ali S., Lin R.Q., Seyni B., Issa G., Zhu X.Q. (2008). Genetic characterisation of Fasciola samples from different host species and geographical localities revealed the existence of *F. hepatica* and *F. gigantica* in Niger. Parasitol. Res..

[bb0020] Andrews S.J., Dalton J.P. (1999). The life cycle of *Fasciola hepatica*. Fasciolosis.

[bb0025] Ardo M.B., Aliyara Y.H., Lawal H., Barkindo A.A. (2013). Economic assessment of bovine fasciolosis in some selected abattoirs of Adamawa state, Nigeria. Intern. J. Livest. Res.

[bb0030] Arias M., Lomba C., Dacal V., Vázquez L., Pedreira J., Francisco I., Piñeiro P., Cazapal-Monteiro C., Suárez J.L., Díez-Baños P., Morrondo P. (2011). Prevalence of mixed trematode infections in an abattoir receiving cattle from northern Portugal and north-west Spain. Vet. Rec..

[bb0035] Bargues M.D., Mas-Coma S. (2005). Reviewing *lymnaeid* vectors of fascioliasis by ribosomal DNA sequence analyses. J. Helminthol..

[bb0040] Bernardo C.C., Carneiro M.B., Avelar B.R., Donatele D.M., Martins I.V.F., Pereira M.J.S. (2011). Prevalence of liver condemnation due to bovine fasciolosis in Southern Espírito Santo: temporal distribution and economic losses. Rev. Bras. Parasitol. Vet..

[bb0045] Biu A.A., Ahmed M.I., Mshelia S.S. (2006). Economic assessment of losses due to parasitic diseases common at the Maiduguri abattoir, Nigeria. African Sci..

[bb0050] Cadmus S.I., Adesokan H.K. (2009). Causes and implications of bovine organs/offal condemnations in some abattoirs in Western Nigeria. Trop. Anim. Health Prod..

[bb0055] CBN (2015). Central Bank of Nigeria Monthly Exchange rates. http://www.cbn.org.ng/rates/ExchRate.asp.

[bb0060] Cheesbrough M. (1999). District Laboratory Practice in Tropical Countries' Part 1, Low price edition.

[bb0065] Damwesh S.D., Ardo M.B. (2012). Epidemiological studies of bovine fasciolosis in Yola modern abattoir, Adamawa state, Nigeria. Vom J. Vet. Sci..

[bb0070] Dean A.G., Sullivan K.M., Soe M.M. (2009). OpenEpi: Open source epidemiologic statistics for public health, Version 2.3.1. http://www.openepi.com/OE2.3/Menu/OpenEpiMenu.htm.

[bb0075] Dohoo I., Martin W., Studahl H., McPike S.M. (2009). Measures of association. Veterinary Epidemiologic Research.

[bb0080] Ejeh E.F., Paul B.T., Lawan F.A., Lawal J.R., Ejeh S.A., Hambali I.U. (2015). Seasonal prevalence of bovine fasciolosis and its direct economic losses (del) due to liver condemnation at Makurdi abattoirs north-central Nigeria. Sokoto J.Vet. Sci..

[bb0085] Fatima M.A., Chislti M.Z. (2008). Report of *Fasciola gigantica* Cobboid, Parasitic trematode in ruminant. Proceedings of 2^nd^ Jammu Kashmir Science Conference, Pakistan.

[bb0090] Gretera H., Batil A.A., Alfaroukh I.O., Grimm F., Ngandolo B.N., Keisera J., Utzingera J., Zinsstag J., Hattendorf J. (2016). Re-infection with *Fasciola gigantica* 6-month post-treatment with triclabendazole in cattle from mobile pastoralist husbandry systems at Lake Chad. Vet. Parasitol..

[bb0095] Hammond J.A. (1965). Observations on fasciolosis in Tanganyika. Bull. Epiz. Dis. Africa..

[bb0100] Ibironke A.A., Fasina F.O. (2010). Socio-economic implications of bovine liver rejection in a major abattoir in south western Nigeria. Rev. Ciˆencias Agr'arias.

[bb0105] Idris H.S., Madara A.A. (2005). Vector competence and prevalence of *Fasciola gigantica* in cattle slaughtered in Gwagwalada abattoir, Abuja, Nigeria. Biol. Environ. Sci. J. Trop..

[bb0110] Jean-Richard V., Crump L., Abicho A.A., Naré N.B., Greter H., Hattendorf J., Schelling E., Zinsstag J. (2014). Prevalence of Fasciola gigantic infection in slaughtered animals in South-Eastern Lake Chad area in relation to husbandry practices and seasonal water levels. BMC Vet. Res..

[bb0115] Kanyari P.W.N., Kagira J.M., Mhoma J.R.L. (2010). Prevalence of endoparasites in cattle within urban and peri-urban areas of Lake Victoria Basin, Kenya with special reference to zoonotic potential. Sci. Parasit..

[bb0120] Khaitsa M.L., Hammond J.A., Opuda-Asibo J. (1994). Use of meat inspection records in veterinary planning. Bull. Epizoot. Dis. Afri..

[bb0125] Kuchai J.A., Chishti M.Z., Zaki M.M., Dar Muzaffar R.S.A., Ahmad J., Tak H. (2011). Some epidemiological aspects of fascioliasis among cattle of Ladakh. Global Vet..

[bb0130] Magaji A.A., Ibrahim K., Salihu M.D., Saulawa M.A., Mohammed A.A., Musawa A.I. (2014). Prevalence of fascioliasis in cattle slaughtered in sokoto metropolitan abattoir, Sokoto, Nigeria. Adv. Epid..

[bb0135] Mas-Coma S. (1997). Secondary reservoir role of domestic animals other than sheep and cattle in fasciolosis transmission in northern Bolivian, Altiplano. Res. Rev. Parasitol..

[bb0140] Mas-Coma S., Bargues M., Valero M. (2005). Fascioliasis and other plant-borne trematoda zoonoses. Int. J. Parasitol..

[bb0145] Mas-Coma S., Valero M.A., Bargues M.D. (2009). Fasciola, lymnaeids and human fascioliasis, with a global overview on disease transmission, epidemiology, evolutionary genetics, molecular epidemiology and control. Adv. Parasitol..

[bb0150] MLFD (2015). Estimated livestock population in Niger State. 2014 Annual Livestock Report of the Ministry of Livestock and Fisheries Development (MLFD), Minna, Niger State, Nigeria, February, 2014.

[bb0155] Njoku-Tony R. (2011). Bovine fascioliasis among slaughtered bovines in selected abattoirs in Imo state, Nigeria. World Rur. Observ.

[bb0160] NPC (2006). Census data for the 2006 national census. The National Population Commission (NPC) of Nigeria, Abuja.

[bb0165] Obadiah S.E. (2010). Preliminary studies on fascioliasis in cattle slaughtered at Jalingo abattoir, Taraba state, Nigeria. Nigerian J. Sci. Techn. Environ. Edu..

[bb0170] Phiri A.M. (2006). Common conditions leading to cattle carcass and offal condemnations at 3 abattoirs in the western province of Zambia and their zoonotic implications to consumers. J. S. Afr. Vet. Assoc..

[bb0175] Phiri A.M., Phiri I.K., Sikasunge C.S., Moirad I. (2005). Prevalence of fasciolosis in Zambian cattle observed at selected abattoirs with emphasis on age, sex and origin. J. Vet. Med..

[bb0180] Qureshi A.W., Tanveer A., Maqbool A., Niaz S. (2012). Seasonal and monthly prevalence pattern of fascioliasis in buffaloes and its relation to some climatic factors in north eastern areas of Punjab, Pakistan. Iranian J. Vet. Res..

[bb0185] Rapsch C., Schweizer G., Grimm F., Kohler L., Bauer C., Deplazes P., Braun U., Torgerson P. (2006). Estimating the true prevalence of *Fasciola hepatica* in cattle slaughtered in Switzerland in the absence of an absolute diagnostic test. Int. J. Parasitol..

[bb0190] Regassa A., Woldemariam T., Demisie S., Moje N., Ayana D., Abunna F. (2012). Bovine fasciolosis: coprological, abattoir survey, and financial loss due to liver condemnation in Bsihoopin municipal abattoir, central Ethiopia. European J.Biol. Sci..

[bb0195] Schillhorn Van Veen T.W. (1997). Sense or nonsense? Traditional methods of animal parasitic disease control. Vet. Parasitol..

[bb0200] Schweitzer G., Braun U., Deplazes P., Torgerson P.R. (2005). Estimating the financial losses due to bovine fasciolosis in Switzerland. Vet. Rec..

[bb0205] Shahzad A., Iqbal Z., Ali M., Chaudhry H.R., Sial N., Ahsan U. (2014). Seasonal prevalence of *Fasciola hepatica* infection in buffaloes of Bahawalpur district of Punjab, Pakistan. J. Infect. Mol. Biol.

[bb0210] Sissay M.M., Uggla A., Waller P.J. (2007). Prevalence and seasonal incidence of nematode parasites and flukes infections of sheep and goats in eastern Ethiopia. Trop. Anim. Health Prod..

[bb0215] Soulsby F.J.L. (1982). Helminths, Arthropoda and Protozoa of Domestic Animals.

[bb0220] Ulayi B.M., Umaru-Sule B., Adamu S. (2007). Prevalence of *Dicrocoelium hospes* and *Fasciola gigantica* in cattle at slaughter in Zaria. Nigerian J. Anim. Vet. Adv..

[bb0225] Valero M.A., Perez-Crespo I., Periago M.V., Khoubbane M., Mas-Coma S. (2009). Fluke egg characteristics for the diagnosis of human and animal fascioliasis by *Fasciola hepatica* and *F. gigantic*. Acta Trop..

[bb0230] Walker S.M., Makundi A.E., Namuba F.V., Kassuku A.A., Keyyu J., Hoey E.M. (2008). The distribution of *Fasciola hepatica* and *Fasciola gigantica* within southern Tanzania - constraints associated with the intermediate host. Parasit..

[bb0235] WHO (1995). Control of food-borne trematode infections. World Health Organization Technical Report, WHO, Geneva, Series 849.

